# Increasing the Number of SNP loci does not Necessarily Improve Prediction Power at Least in the Comparison of *MTHFR* SNP and Haplotypes

**DOI:** 10.2188/jea.JE2008022

**Published:** 2008-12-17

**Authors:** Koichi Miyaki, Yoshimitsu Takahashi, Yixuan Song, Ling Zhang, Masaaki Muramatsu, Takeo Nakayama

**Affiliations:** 1 Preventive Medicine for Cerebrovascular Disease, Department of Neurology, Shool of Medicine, Keio University, Tokyo, Japan; 2 Department of Health Informatics, School of Public Health, Graduate School of Medicine, Kyoto University, Kyoto, Japan; 3 Department of Molecular Epidemiology, Medical Research Institute, Tokyo Medical and Dental University, Tokyo, Japan; 4 Department of Clinical Research and Informatics, Research Institute, International Medical Center of Japan, Tokyo, Japan

**Keywords:** Folic Acid, Randomized Controlled Trials, Methylenetetrahydrofolate Reductase (MTHFR), Haplotypes, Informativeness

## Abstract

**Background:**

Rapid advances in genotyping technology have made it possible to easily utilize a large number of genetic markers. According to information theory, an increase in the number of markers provides more information; however, the clinical usefulness does not increase linearly. This study aimed to assess the effect of folic acid supplementation quantitatively in *MTHFR* haplotypes, and compare its prediction power with that of the C677T single nucleotide polymorphism (SNP) alone.

**Methods:**

The study was a randomized, double-blind, placebo-controlled trial, designed in accordance with the CONSORT statement. The participants were 202 healthy Japanese males who were administered either folic acid at 1 mg/day or a placebo postoperatively for 3 months. The primary endpoint was the total plasma homocysteine levels (tHcy). Stratified analysis by HapMap-based tag SNPs was performed.

**Results:**

Of 52 SNPs on the *MTHFR* gene, 4 SNP loci covering more than 80% of the information were selected, and the haplotypes were estimated. The haplotypes were classified into 3 groups (Hap0, Hap1, and Hap2), on the basis of the number of times the most frequent haplotype was present. The greatest decrease was observed in Hap2 (6.61 µmol/L), compared with the other haplotypes (Hap0, 2.67; Hap1, 2.60) (trend test, *P* < 0.01). The haplotype information obtained was not more informative than that obtained with grouping by a single SNP, C677T, which strongly influences enzyme activity.

**Conclusions:**

Grouping by the C677T SNP alone was almost as good a predictor of the homocysteine-lowering effects as was grouping by the 4 best SNPs. This shows that increasing the number of typed SNPs does not necessarily provide more information, at least for this gene. A more efficient, cost-informative method for analyzing genomic data is required.

## INTRODUCTION

Single nucleotide polymorphisms (SNPs), the most common type of polymorphisms in the human genome, are so common that the information they carry seems highly redundant in high linkage disequilibrium (LD) regions of the human genome.^1^ By selecting a small fraction of SNPs, i.e., the tag SNPs, it is possible to significantly reduce the genotyping efforts, with the loss of only a small amount of predictive power.^2^ The International HapMap project-a collaboration between academic, public, and private institutions from the USA, Japan, China, Nigeria, UK, and Canada-has expanded our knowledge of LD patterns in the human genome.^3^

Recently, homocysteine-lowering intervention for stroke prevention has received increasing attention, and randomized controlled trials and meta-analysis of the same have been conducted.^4^^-^^6^ It has been suggested that high homocysteine levels are a modifiable, independent risk factor for coronary artery disease, stroke, and deep vein thrombosis.^7^^-^^9^

We decided to focus on the methylenetetrahydrofolate reductase (*MTHFR*) gene-a key enzyme in homocysteine metabolism-as a representative of functional polymorphism. Individuals homozygous for the T allele of the *MTHFR* C677T polymorphism have higher plasma homocysteine levels and show a higher response rate to folic acid than individuals with other genotypes.^10^ Cumulative meta-analysis suggests that this polymorphism shows a dose-response association with the risk of ischemic stroke.^11^ The observed increase in the risk of stroke among individuals homozygous for the *MTHFR* T allele is similar to that predicted from the difference in homocysteine concentration conferred by this variant.^12^ This concordance suggests a causal relationship between homocysteine and stroke, on the basis of Mendelian randomization.

An approach known as a genome-wide association study (GWA Study) has emerged in the UK and US. This approach involves screening markers across the complete genomes of many individuals to find genetic variations associated with a particular disease. These studies have identified gene variations that are strongly associated with obesity, cardiovascular disease, coronary artery disease, and several other diseases.^13^^-^^14^ The whole-genome approach is expected to be useful in identifying new genetic associations in the future. Due to the high cost involved in the whole-genome approach, it is reasonable to analyze the efficacy of these studies.

Rapid advances in genotyping technology have made it possible to utilize a large number of genetic markers more easily. According to information theory, more markers provide more information; however, the clinical usefulness does not appear to increase linearly.

Here, we focused on the *MTHFR* C677T SNP, which has been shown to be a determinant of the homocysteine level and folic acid responsiveness, and we hypothesized that adding another SNP via an informatics approach may predict the response to folic acid more effectively. This study assessed the effects of folic acid supplementation quantitatively in each estimated haplotype, and compared the prediction power with that of the C677T SNP alone.

## METHODS

### Study participants and design

A total of 326 healthy Japanese men, who worked at a corporation in Kanagawa Prefecture, participated in this study. These participants were a part of our previous occupational cohort study for exploring the association of lifestyle factors with atherosclerosis, on the basis of different genetic factors in the Japanese population.^10^ We calculated the sample size according to the results of our pilot trial. We estimated the standard effect size in each genotype, i.e., CC, CT, and TT, to be 0.5, 0.55, and 0.75, respectively, by the serum folate stratified analysis of the cross-sectional data in the pilot study. The planned enrolment of 68, 56, and 32 subjects of each genotype facilitated the detection of pairwise differences with 80% power, with a 2-sided test at α = 0.05. We obtained written informed consent from 210 genetically unrelated healthy males for participation in the intervention study. After exclusion of those who were consuming folic acid or drugs known to affect folic acid metabolism, the subjects in this trial subsequently consisted of 203 healthy males.

The study was designed around genotype-stratified, randomized, double-blind, placebo-controlled trials. Intervention consisted of folic acid at 1 mg/day or an identical-looking placebo for 3 months, since a minimum dosage of 0.8 mg/day has been reported as necessary to achieve the maximum reduction in the plasma total homocysteine (tHcy) level.^15^ The endpoint was the change in the tHcy level. All measurements were conducted at the baseline, 1 month, and 3 months after the initiation of the intervention. Subjects were randomly allocated to the folic acid group and placebo group at a ratio of 1:1, according to random allocation sequence by the SAMPSIZE ver.2 software (Blackwell Science, London, UK).

The doctors, nurses, and technicians in the clinic had no information regarding the group assignment. The subjects and study design were originally determined for our previous study, and this study was conducted as an ancillary analysis.

### Measurements

We sampled approximately 15 mL of whole blood from each participant, using 1 auto-separation tube, 2 citrate tubes, and 1 plain tube, in accordance with the informed consent form. Age and smoking status were self-reported, and medical history was acquired via interviews. Height, weight, systolic and diastolic blood pressures, fasting blood glucose level, serum lipid levels (total cholesterol, triglyceride, and high-density lipoprotein [HDL] cholesterol), aspartate aminotransferase (AST), alanine aminotransferase (ALT), gamma-glutamyl transpeptidase (gamma-GTP), blood urea nitrogen (BUN), serum creatinine (Cr), and serum uric acid (UA) levels were measured for all the subjects. Fasting blood samples were obtained, and plasma for tHcy determination was separated with minimal delay and stored at -20°C until analysis. The tHcy levels were assayed using the high-performance liquid chromatography (HPLC) method.^16^ The validity of all the measurements conducted in the laboratory was certified by the College of American Pathologists, USA.

To monitor the intake of folic acid or placebo capsules, we distributed a diary every month (3 times) for each participant to report the actual daily intake, and measured the serum folate levels at the baseline, 1 month, and 3 months after the initiation of the intervention.

The ensemble database (http://www.ensembl.org) was used to identify an initial set of 52 SNPs across a 193-kb region of the *MTHFR* gene on chromosome 1 (Location, 1p36.3). Of these 52 SNPs, 9 had a minor allele frequency of more than 15% in the Japanese population, based on the HapMap database (http://hapmap.org/), and 4 SNPs [rs1801131 C/A, rs9651118 T/C, rs1476413 G/A, and C677T] were selected by the Tagger algorithm (r2 > 0.8).^17^

DNA was extracted from whole blood using the phenol/chloroform method. Three SNPs (rs1801131 C/A, rs9651118 T/C, and rs1476413 G/A) were genotyped by polymerase chain reaction (PCR) followed by melting curve analysis using a LightCycler 480 (Roche Diagnostics, Penzberg, Germany). The primer and probe sequences are shown in Table 1. PCR was performed with 0.5 µM of the sense primer and 0.05 µM of the antisense primer in a reaction mixture containing 0.2 µM anchor probe, 0.2 µM sensor probe, 10 ng dried-down DNA, 3.0 µM MgCl_2_, 0.5 µL 10 × PCR buffer, 0.2 mM dNTP, and 0.1 U FastStart DNA Polymerase (Roche Diagnostics Inc.) in a total volume of 5 µL. The cycling program consisted of 5 min of initial denaturation at 95°C and 55 cycles of denaturation at 95°C for 10 s, annealing at 60°C for 10 s, and extension at 72°C for 10 s. After the completion of PCR, melting curve analysis was performed by heating the mixture at 95°C for 60 s and then keeping it at 40°C for 60 s. The plate was then heated from 40°C to 80°C by a gradient of 0.1°C per second. Melting curve data were collected and classified using the LightCycler Genotyping software, and the genotypes were determined. Genotyping for *MTHFR* C677T polymorphism was determined by PCR followed by *Hinf*I (TaKaRa Bio Inc., Shiga, Japan) restriction digestion, as previously described. All the measurements for this study are available from our previous study.^10^

**Table 1.** Details of the Loci and haplotypes in the *MTHFR* gene

	Locus 1	Locus 2	Locus 3	Locus 4	
Selected tag SNP	C677T	A1298C	**—**	**—**	
dbSNP ID	rs1801133	rs1801131	rs9651118	rs1476413	
Allele	C/T	A/C	T/C	G/A	
Peptide allele	A/V222	E429A	intron	intron	
Positionb (bp)	11,778,965	11,177,742	11,796,480	11,786,566	
MAF ^†^	0.364	0.178	0.422	0.205	


Haplotype					Frequency
#1	T	A	T	G	0.39906
#2	C	A	C	G	0.32846
#3	C	C	T	A	0.18728
#4	C	A	T	G	0.05451
#5	T	A	C	G	0.01072
#6	C	C	T	G	0.01006
#7	C	A	C	A	0.00432
#8	C	C	C	A	0.00427
#9	T	A	C	A	0.00133

† MAF : Minor allele frequency from the International HapMap Database

### Haplotype estimation

To infer haplotypes consisting of the 4 abovementioned SNPs, we used the estimation maximization algorithm in the haplo.stat package (Jason P. Sinnwell and Daniel J. Schaid of the Mayo Foundation for Medical Education and Research), which is written in the R language.

Haplotypes were estimated from 4 loci: locus 1 (C677T), locus 2 (rs1801131 C/A), locus 3 (rs9651118), and locus 4 (rs1476413). On the basis of the Tagger algorithm, these SNPs were the 4 best SNPs among 19.3 kbp, which is the entire length of the *MTHFR* gene.^17^ Table 1 shows the details of the loci and haplotypes. The TATG haplotype (Haplotype #1) consisted of a T allele at locus 1, an A at locus 2, a T at locus 3, and a G at locus 4. The TATG haplotype appeared most frequently (39.9%). All the participants were classified into 3 groups (Hap0, Hap1, and Hap2) according to the number of times the most frequent haplotype, i.e., the TATG haplotype, was present. Individuals of the Hap0 group had no TATG haplotype; individuals of the Hap1 group had 1 TATG haplotype; and individuals of the Hap2 group had 2 TATG haplotypes.

### Statistics

A Student’s *t* test or the Welch *t* test was applied to compare variables between groups after Levine’s test for equality of variance. Differences in the plasma homocysteine levels among haplotype groups (multiple comparisons) were assessed using Tukey’s post hoc test of analysis of variance (ANOVA). Odds ratios (ORs) and 95% confidence intervals (95% CIs) were calculated using logistic regression analysis. All statistical analyses were performed using SPSS for Windows version 14.0J (SPSS Inc., IL, USA). Statistical significance was accepted for a 2-tailed *P*-value of <0.05.

### Ethics

The present study was approved by the ethical committee of Keio University School of Medicine and the Ethics Review Committee of the Medical Research Institute, Tokyo Medical and Dental University. Written informed consent was obtained from each participant.

## RESULTS

During the 3-month intervention period, 11 out of 210 participants were lost to follow-up, among whom 4 were in the folic acid group and 7, in the placebo group. The overall follow-up rate was 94.6%.

Of the 203 participants who were analyzed, 1 individual’s haplotype could not be estimated because a DNA sample was not available, so he/she was eliminated from this study. Of the 101 people who were allocated to the folic acid group, 35 were in Hap0; 49, in Hap1; and 17, in Hap2. Of the 101 people who were allocated to the placebo group, 35 were in Hap0; 49, in Hap1; and 17, in Hap2. The *P* value for the Hardy-Weinberg equilibrium by the exact test is 0.885549.

The baseline characteristics of the participants in each haplotype group, for both folic acid and the placebo, are shown in Table 2.

**Table 2.** Baseline characteristics in each haplotype group

	Number of TATG Haplotypes											

	0 (Hap0)				1 (Hap1)				2 (Hap2)			
		
	Folic acid group		Placebo group		Folic acid group		Placebo group		Folic acid group		Placebo group	
	(n = 35)		(n = 35)		(n = 49)		(n = 49)		(n = 17)		(n = 17)	
Demographics												
Age (years)	45.6	(11.3)	45.7	(12.4)	45.1	(12)	46.6	(11)	45.2	(10.1)	48.4	(11.4)
Gender (male/female)	35/0		35/0		49/0		49/0		17/0		17/0	
Body-mass index (kg/m^2^)	24.4	(5)	23.2	(2.9)	23.9	(3.3)	23.7	(3.5)	23.1	(3.2)	22.7	(3.8)
Smoking	17	(48.6%)	20	(57.1%)	29	(59.2%)	30	(61.2%)	10	(58.8%)	12	(70.6%)
Alcohol intake (g/week)	186.7	(187)	129.2	(179)	120.7	(177.3)	171.7	(214.4)	322.4	(303.6)	134.2	(152)
Blood pressure												
Systolic (mmHg)	136.4	(19.1)	132.3	(14.4)	137.3	(20.2)	131.5	(19.1)	135.8	(13.6)	133.6	(17.3)
Diastolic (mmHg)	83.4	(12.1)	78.2	(10.1)	83.8	(14.5)	81.2	(13)	85.6	(13.9)	81.4	(12.4)
Laboratory values												
Total cholesterol (mg/dL)	214.7	(34.8)	203.4	(38.6)	207.5	(42.5)	200.7	(37.8)	208.8	(41.4)	210.8	(31.3)
Triglyceride (mg/dL)	151.6	(98.3)	159.9	(104.7)	125.3	(65.4)	147.4	(95.6)	110.8	(53)	120.8	(46.4)
HDL cholesterol (mg/dL)	58.3	(15.6)	50.4	(11)	53.8	(12.5)	53.0	(10.2)	61.8	(17.1)	53.5	(16.4)
AST/GOT (IU/L)	24.1	(7.5)	23.5	(7.9)	25.1	(9.1)	27.7	(21)	24.4	(5.8)	21.6	(4.1)
ALT/GPT (IU/L)	29.3	(17.3)	26.1	(16.3)	32.3	(19.7)	33.4	(32)	24.2	(10.5)	22.4	(9.8)
γ-GTP (IU/L)	61.2	(60.4)	43.5	(44.3)	65.4	(53.2)	50.0	(41.1)	53.0	(37.6)	35.5	(26.6)
Blood urea nitrogen (mg/dL)	13.8	(3.4)	15.3	(2.3)	14.4	(2.8)	13.8	(2.4)	13.4	(2.8)	13.7	(3.1)
Serum creatinine (mg/dL)	0.87	(0.1)	0.90	(0.1)	0.83	(0.1)	0.88	(0.1)	0.82	(0.1)	0.82	(0.1)
Serum uric acid (mg/dL)	5.84	(1.1)	5.74	(1.1)	5.53	(1.1)	5.68	(1.2)	5.72	(1.3)	5.54	(1)
Serum Vitamin B12 (pg/mL)	650.0	(460.9)	521.2	(147.5)	501.9	(158.3)	513.6	(254.5)	483.8	(134.8)	558.1	(162.4)
Serum folic acid (ng/mL)	6.43	(2.3)	7.07	(3.1)	6.63	(3.5)	7.44	(5.2)	5.67	(2.7)	5.78	(2.3)
Outcome index												
Plasma homocysteine (*µ* mol/L)	9.73	(2.9)	8.85	(1.7)	9.60	(1.8)	9.40	(2.2)	14.7	(9.3)	17.2	(13.2)
hsCRP (mg/dL)	1.07	(1.9)	0.85	(0.9)	1.18	(1.9)	1.07	(2)	0.67	(0.8)	0.92	(1.2)
baPWV (cm/s)	1410	(227.3)	1357	(226.1)	1409	(241.6)	1398	(213.7)	1385	(158.6)	1346	(252.1)
ABI	1.14	(0.09)	1.13	(0.09)	1.11	(0.07)	1.11	(0.08)	1.15	(0.07)	1.12	(0.08)

The serum folic acid levels in the folic acid group were significantly higher than those in the placebo group at both 1 month and 3 months after the initiation of the intervention (for both, *P* < 0.01), confirming that this group did consume folic acid. The plasma tHcy levels in the folic acid group were significantly lower than those in the placebo group at both 1 month and 3 months (for both, *P* < 0.01).

In the folic acid group, the serum folic acid level in the Hap2 group was significantly lower than those in the Hap0 and Hap1 groups at both 1 month and 3 months (for both, *P* < 0.01). In the placebo group, the serum folic acid level of the Hap2 group was not significantly different from those of the Hap0 and Hap1 groups at 1 month (*P* = 0.13).

In addition to the significant increase in folic acid and significant decrease in tHcy, there was also a significant difference between the 3 haplotypes in the folic acid group. As shown in Table 3, the Hap2 group showed the largest decrease in tHcy.

**Table 3.** Changes in the outcome variables in each haplotype group

	Number of TATG Haplotype					

	0 (Hap0)		1 (Hap1)		2 (Hap2)	
		
	Folic acid group	Placebo group	Folic acid group	Placebo group	Folic acid group	Placebo group
	(n = 35)	(n = 35)	(n = 49)	(n = 49)	(n = 17)	(n = 17)
Change in plasma homocysteine (µmol/L) ^†^						
After 1 month	-2.67 (2.29)**	-0.40 (1.65)	-2.60 (1.68)**	-1.02 (1.18)	-6.61 (6.42)	-2.09 (6.96)
After 3 months	-3.13 (2.13)**	-0.90 (1.51)	-2.82 (1.27)**	-0.83 (1.40)	-6.84 (8.42)*	4.87 (16.2)
Change in serum folic acid (ng/mL) ^†^						
After 1 month	12.9 (5.35)**	0.23 (2.25)	11.5 (6.4)**	-0.08 (4.23)	9.51 (6.67)**	0.16 (1.33)
After 3 months	13.5 (4.47)**	-0.38 (2.45)	15.0 (4.84)**	-1.19 (4.74)	11.07 (6.54)**	-0.49 (2.22)

† mean (SD) is indicated.

** p< 0.01 and *p < 0.05 for t test versus placebo group.

Changes are evaluated from the baseline level in each group.

**Figure 1.**
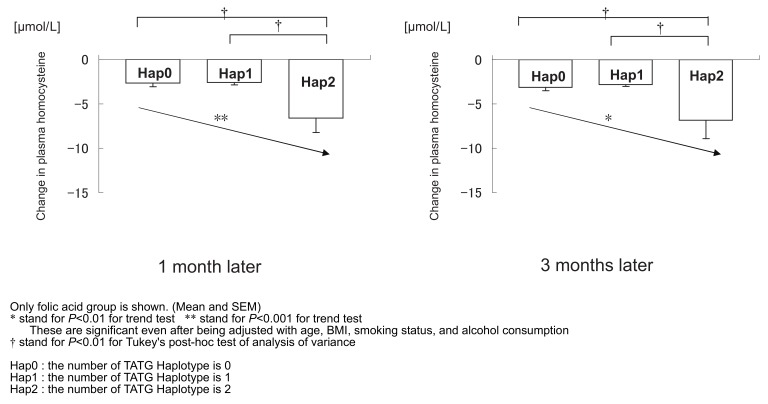
Differences between haplotype groups in the decrease of homocysteine

The significant differences in the decrease of plasma tHcy between the haplotypes of the folic acid group are shown in Figure 1. Hap2 showed the largest decrease in plasma tHcy at both 1 month and 3 months after the initiation of the intervention. The effective reduction in the tHcy levels in the Hap2 group was estimated to be 2.32-fold compared with the Hap0 and Hap1 groups. Significant linear trends were observed between the number of haplotypes and decrease in plasma tHcy at 1 month and 3 months (for both, *P* < 0.01). Both results obtained from the trend tests were statistically significant, even after adjusting for age, body mass index (BMI), smoking status, and alcohol consumption at 1 month and 3 months (for both, *P* < 0.01). Tukey’s post hoc test of ANOVA demonstrated a statistically larger decrease in the Hap2 group than in the Hap0 and Hap1 groups (for both, *P* < 0.01 at 1 month and 3 months).

## DISCUSSION

**Figure 2.**
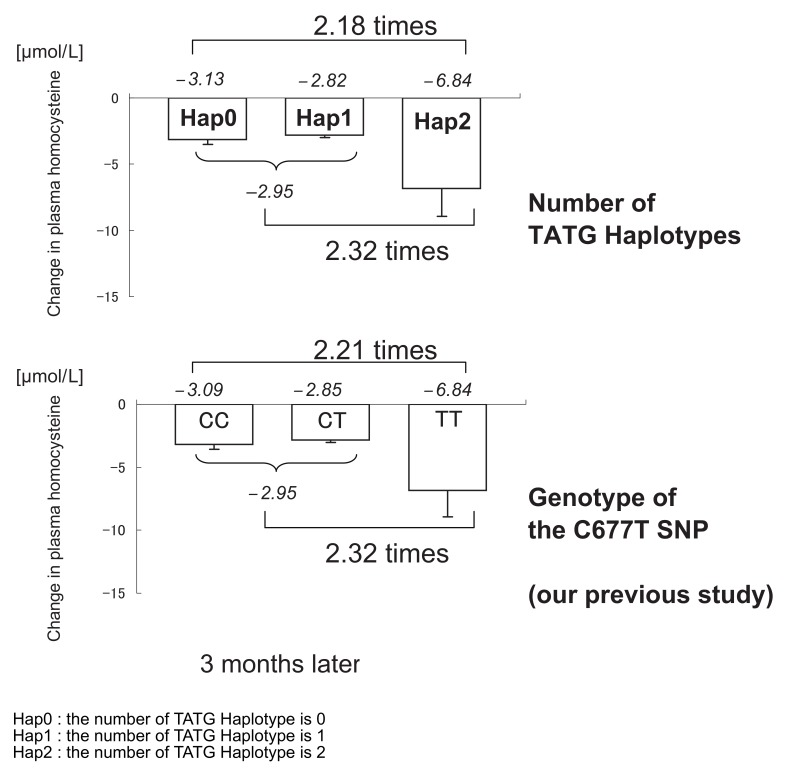
Comparison of the homocysteine reduction between TATG Haplotype group and C677T SNP

All the haplotypes in the folic acid group showed a significant decrease in the plasma tHcy levels. The degree of reduction from the baseline was almost the same at 1 month and 3 months after the initiation of intervention. This indicates that the tHcy lowering effect of folic acid supplementation at 1 mg/day begins to plateau after 1 month. We estimated the haplotypes from 4 SNPs, including *MTHFR* C677T (Table1), and we report that as a result of folic acid supplementation, the Hap2 group attained a 2.3-fold beneficial decrease in the tHcy levels compared with the subjects with the Hap0 and Hap1 groups. Our previous study reported that the subjects homozygous for the *MTHFR* C677T minor allele attained a 2.3-fold beneficial decrease in the tHcy levels compared with those homozygous for the major allele. The significant reduction in the tHcy levels in the Hap2 group compared with those in the Hap0 and Hap1 groups was equal to the reduction in the tHcy levels in the subjects with the TT genotype compared with those with the CT and CC genotypes (Figure 2).

On comparing the haplotypes with *MTHFR* C677T genotypes, we found that in the folic acid group, 34 of 35 individuals in the Hap0 group had the *MTHFR* C677T CC genotype and 1 had the CT genotype, all 49 individuals in the Hap1 group had the CT genotype, and all 17 individuals in the Hap2 group had the TT genotype. In the placebo group, 34 of 35 individuals in the Hap0 group had the CC genotype at this locus and 1 had the CT genotype, all 49 individuals in the Hap1 group had the CT genotype, and all 17 individuals in the Hap2 group had the TT genotype. Grouping according to the *MTHFR* C677T genotype was similar to grouping according to haplotypes. Since the effect of *MTHFR* C677T was determinately stronger than that of the other tag SNPs in this study, we have not provided new evidence of tHcy reduction.

This study demonstrates the same findings regarding the tailor-made prevention of atherosclerosis using folic acid supplementation, and is as informative as our previous study. We interpreted our results with the help of several studies that have been reported. Informative tag SNPs have been continuously reported, and the informativeness of tag SNPs selected by certain methods has been estimated.^1^^,^^18^^,^^19^ As Liu et al. measured informativeness, they found that the inclusion of less informative markers might add noise and worsen the results.^19^ This suggests that the 3 additional SNPs reported in this study might be less informative than *MTHFR* C677T. Nicolas et al. verified the information content^20^ measured in bits versus the number of tag SNPs selected by certain methods, and justified the selection of tag SNPs on the basis of haplotype informativeness.^1^ Although we used this method based on informativeness, it did not provide informative results. Furthermore, as cost-effective SNP genotyping was mentioned, tagging quality assessment and optimization, that is, minimizing the number of tag SNPs, were included as a step in the selection of tag SNPs.^21^

According to the GWA study, 24 independent association signals were identified by examining a 500K Mapping Array Set of 14,000 cases of 7 common diseases and 3,000 shared controls.^13^ The Framingham Heart Study 100K Project analyzed genome-wide SNP associations for blood pressure and arterial stiffness to examine the 100K Mapping Array Set of the Framingham Heart Study families. However, neither report identified independent association signals with hypertension. Phenotypic studies require multigenetic factors as well as environmental factors and interactions among these factors, which make them unsuitable for generating appropriate predictions and hypotheses. Moreover, given the substantial cost of the GWA study, it is unlikely to be cost-effective.^22^ The fact that these whole-genome approaches were more effective than other approaches, e.g., the public health approach, might be controversial. In our study, additional typing of 3 tag SNPs and haplotype estimation were required. Although the cost of conducting this study and the data required was greater than those of our previous study, the results were only as informative and, therefore, less “cost-informative” than our previous study.

The analysis of whole genomes and large numbers of SNPs is increasing at an explosive rate. We believe that it is important to analyze multiple SNPs and to explore SNPs related to diseases, especially multifactorial disorders. This study demonstrates that increasing the data volume of SNPs does not necessarily lead to meaningful information. Moreover, considering the limited workload and cost, we may need to suppress the increase in the analysis of multiple SNPs when discussing “cost-informativeness.”

### Limitations

This study had some limitations. The participants were 202 healthy Japanese men who worked at a corporation in Japan, so this was a relatively small cohort size. Despite this, the results were statistically significant. The size was statistically determined for assessing the effect of folic acid supplementation quantitatively in each *MTHFR* C677T genotype, and it was determined to be significant.

The participants consisted only of Japanese men. Little difference was observed in the allele frequencies among some ethnicities according to the HapMap database. The plasma tHcy levels were different for both sexes. The effect on the tHcy levels in women was not examined in this study, but the difference in the effect between men and women should be assessed separately. We think that the participants were appropriately limited.

Our current analysis does not include the folic acid intake level, which is known to affect the homocysteine level. Instead of the folic acid intake level, we used the serum folic acid level, which is known to be well associated with the folate intake level,^23^^,^^24^ and provides a better quantitative value than the intake estimation.

All the SNPs were not analyzed. We conducted the haplotype analysis based on 4 tag SNPs, which were effectively selected according to the optimal method described in a study^15^ that was based on a biological mechanism. As the SNPs covered over 80% of the information, we believe that the method of selection did not result in a large difference with the whole-genome approach.

### Conclusion

In the 3 groups (Hap0, Hap1, and Hap2) chosen by the number of times the most frequent haplotype was present, which was estimated to be 4 SNP loci from 52 SNPs, the plasma tHcy levels in Hap2 (6.61 µmol/L) significantly decreased compared with those in the other haplotypes (Hap0, 2.67; Hap1, 2.60). These results were not more informative than those obtained with grouping by the single SNP, C677T, which strongly influences enzyme activity. The results suggest that increasing the number of SNPs typed does not necessarily provide more information, at least with regard to this gene. To avoid wasting typing costs, we need a more effective way of handling large quantities of genomic data.
